# Risk factors for avian influenza in Danish poultry and wild birds during the epidemic from June 2020 to May 2021

**DOI:** 10.3389/fvets.2024.1358995

**Published:** 2024-02-21

**Authors:** Yangfan Liu, Lene Jung Kjær, Anette Ella Boklund, Charlotte Kristiane Hjulsager, Lars Erik Larsen, Carsten Thure Kirkeby

**Affiliations:** ^1^Department of Veterinary and Animal Sciences, Faculty of Health and Medical Sciences, University of Copenhagen, Frederiksberg, Denmark; ^2^Department of Virus and Microbiological Special Diagnostics, Statens Serum Institut, Copenhagen, Denmark

**Keywords:** avian influenza, disease surveillance, risk factors, wild birds, poultry, epidemic

## Abstract

Exploring the risk factors of avian influenza (AI) occurrence helps us to monitor and control the disease. Since late 2020, the number of avian influenza outbreaks in domestic and wild birds has increased in most European countries, including Denmark. This study was conducted to identify potential risk factors for wild birds and poultry during the epidemic in 2020/2021 in Denmark. Using Danish AI surveillance data of actively surveyed poultry and passively surveyed wild birds from June 2020 to May 2021, we calculated geographical attributes for bird locations and assessed the potential risk factors of AI detections using logistic regression analyses. 4% of actively surveyed poultry and 39% of passively surveyed wild birds were detected with AI circulating or ongoing at the time. Of these, 10 and 99% tested positive for the H5/H7 AI subtypes, respectively. Our analyses did not find any statistically significant risk factors for actively surveyed poultry within the dataset. For passively surveyed wild birds, bird species belonging to the Anseriformes order had a higher risk of being AI virus positive than five other taxonomic bird orders, and Galliformes were of higher risk than two other taxonomic bird orders. Besides, every 1 km increase in the distance to wetlands was associated with a 5.18% decrease in the risk of being AI positive (OR (odds ratio) 0.95, 95% CI 0.91, 0.99), when all other variables were kept constant. Overall, bird orders and distance to wetlands were associated with the occurrence of AI. The findings may provide targets for surveillance strategies using limited resources and assist in risk-based surveillance during epidemics.

## Introduction

There has been an increase in reports of avian influenza (AI) in Europe during recent decades (1). The AI virus (AIV) is classified as highly pathogenic avian influenza virus (HPAIV) or low pathogenic avian influenza virus (LPAIV), and may pose a threat to birds, humans, and other mammals. The viruses are divided into subtypes on the basis of two proteins on the surface: hemagglutinin (HA) and neuraminidase (NA). Previous studies from Europe demonstrated that the persistent detection of different viral subtypes in poultry and wild birds might affect public health (2). Measures taken to control the disease during outbreaks in Denmark included culling of poultry and movement restrictions (3). During the HPAI season in 2020/2021, subtype H5N8 was the dominant strain circulating in poultry and wild birds in European countries (4). LPAIV of the subtypes H5 and H7 were also reported during the years 2020 and 2021 in Europe in Denmark, Belgium, France, Italy, the Netherlands, and the UK (1). These subtypes have previously been described as capable of evolving into HPAIV (e.g., in Italy), and outbreaks with LPAI H5 and H7 viruses are therefore of concern (5, 6).

In the study period between June 2020 and May 2021, Denmark conducted AI surveillance in poultry and wild birds in agreement with Commission Decisions 2005/734/EC, 2006/605/EC, and 2010/367/EU. For wild birds, passive surveillance refers to the EU mandatory surveillance of AIV in dead and moribund birds, where citizens voluntarily report the locations of found birds and the authority selects from the reported birds and performs laboratory diagnosis. For poultry, passive AI surveillance refers to the surveillance of clinical signs and changes in production figures, and active AI surveillance refers to the routine risk-based sampling of healthy birds. A number of inclusion criteria are given for registered farms that require active surveillance in Executive Order No. 1456 of 12/12/2019,^1^ which, among other criteria, include the need for AIV testing before movement or selling. Cycles in poultry production can, therefore, lead to seasonal variation in the number of poultry submissions.

With extensive coastlines and rich wetlands, Denmark provides an ideal habitat for waterfowl and migratory birds such as swans, geese, and gulls (7–9). The distance to wetlands and coastline can be used as a proxy for attractive habitats for waterfowl and migratory pathways of wintering birds, and it is thus relevant for poultry farmers considering farm locations (10). Infectious wild birds can shed virus via saliva and droppings, and susceptible birds may become infected by direct or indirect contact with infectious birds or their secretions (11). A study from the Netherlands found that virus detection in domestic ducks coincided with the prevalence peak in wild ducks, indicating that wild birds might have a role in the transmission of AIV to poultry (12).

Land cover type can be considered a proxy for the variation in wild bird foraging and breeding behavior (13), and it is suspected to result in differences in the spatial pattern of AI occurrence. For instance, studies from Central Asia found that wetland, cropland, and urban land around farms were significant factors in their best-fit model for poultry HPAI H5N1 outbreaks, yet the association was only found between urban land and HPAI H5N1 detection in waterfowl (14). In contrast, land cover was the largest contributor when using a disease distribution model for AI outbreaks in California, United States (15). In Denmark, Kjær et al. (16) investigated landscape effects on the presence of AIV in wild birds and found that coastal areas and wetlands were associated with the detection of AIV. In the Netherlands, a higher density of certain wild bird species was found around farms located in water-rich areas compared to non-water-rich areas, potentially contributing to more effective transmission in these areas (10).

In addition to geographical attributes, other risk factors may facilitate AI outbreaks. In the Netherlands, the spatial distribution of some wild bird species was found to be a predictor of the risk of HPAIV outbreaks in poultry, most notably that of mallards (17). Furthermore, in Australia, Ferenczi et al. (18) found that an increase in temperature during the coldest quarter of the year was associated with an increased risk of domestic HPAIV outbreaks.

Identifying risk factors of AIV transmission using surveillance data during epidemics can help decision-makers determine the optimal surveillance strategy. Denmark faced an unprecedented challenge with increasing numbers of poultry affected by outbreaks of H5 HPAI virus belonging to clade 2.3.4.4b during the epidemic season of 2020/2021 (19), and it is important to assess potential AI risk factors in the Danish bird population during this season. The objective of our study was to investigate the spatial distribution of AI detections with regards to potential risk factors for AI and in particular H5/H7 detection from June 2020 to May 2021 based on data from active surveillance in poultry and from passive surveillance in wild birds.

## Methods

In this study, two data sets with diagnostic results from the period 1 June 2020 to 31 May 2021 were used, based on the active surveillance of poultry (including farm-reared game birds) and passive surveillance of wild birds.

### AI surveillance in Denmark

**Figure 1:**
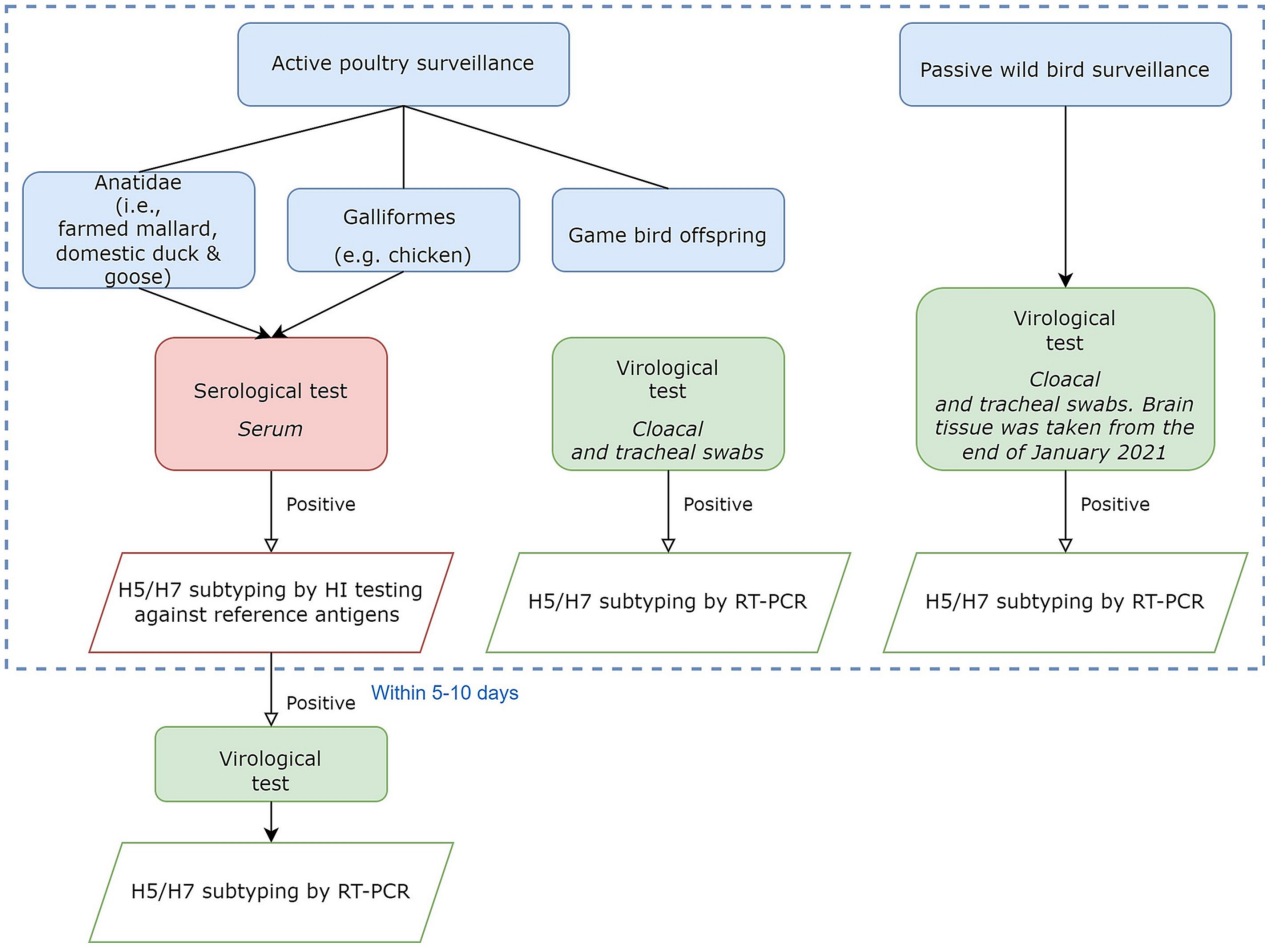
Flowchart showing different laboratory testing procedures to obtain surveillance data. The focus was on the detection of H5 and H7 subtype viruses. Testing results within the dashed blue border were used in the later stages. Further details are in the Supplementary material.

Figure 1 briefly demonstrates the origin of the surveillance data. Risk-based active surveillance was routinely conducted at selected farms registered in Danish Central Husbandry Register (CHR). Wild birds were monitored passively with a focus on AI high-risky target species (20). Positive samples with H5 and H7 were targeted for subtyping and pathotyping.

### Data collection

#### Data structure

We used data from the Danish AI surveillance programs from June 2020 to May 2021. All laboratory tests were performed by the Danish National Reference Laboratory (NRL) for AI, which transferred the results to the Danish Veterinary and Food Administration (DVFA).

We obtained data of the active poultry surveillance from DVFA and information about Danish poultry holdings from CHR, including the geographical coordinates of the holdings and their flock sizes, also provided by DVFA. Based on registered poultry records from CHR, we defined a poultry holding as a farm with a unique identification number and a unique set of coordinates. Each farm may hold multiple species in separate flocks, and each flock might consist of several hierarchically structured sub-flocks. Flock size represents the number of birds belonging to the same species in a farm.

Similarly, data from the passive wild bird surveillance program were obtained from the DVFA. Records of dead wild birds consisted of the sampling dates, species, unique CHR numbers, and testing results.

#### Aggregation to flock/event level

We aggregated all samples in the data to show the presence/absence of AI for the same location, time of testing, and type of bird. We defined the aggregated data as being at flock level for poultry and event level for wild birds. For actively surveyed poultry, individual bird samples were aggregated by unique CHR numbers, species, and sampling dates within 10 days and then months. The aggregation on dates within 10 days was used to avoid double counting introductions in flocks where serological samples were followed up by HI or RT-PCR tests (Figure 1). Except for one submission from a chicken flock that was diagnosed by virological testing rather than serology, active poultry surveillance consisted of virological results for farmed game birds and serological test results for all other poultry. For passively surveyed wild birds, due to a wide variety of species and wild birds’ high similarity in the same order, we use bird orders instead of species. Thus, wild birds were aggregated by coordinates, sampling date, and bird order.

For poultry as well as wild birds, if RT-PCR or serology results identified one or more birds as positive for any AIV subtypes, the flock/event was considered AI positive. Additionally, if one or more birds were found to be H5/H7 positive, the flock/event was considered H5/H7 positive.

#### Analysis preparation

All AI detections were included in our analysis regardless of subtype, and the statistical analyses were therefore conducted on the presence/absence of AI at a location as a whole. For consistency in statistical analysis, we assumed that serological and virological diagnostic results from active poultry surveillance represented the same outcome, for which we used the term AI detection or occurrence to describe a positive result. Additionally, all poultry submissions collected by passive surveillance reported poultry outbreaks on farms due to the epidemic situation in the study period, and 15 poultry outbreaks were detected. We were unable to include passive poultry surveillance data in our study due to the limited sample size of only 15 outbreaks in the study period and the presence of numerous potential risk factors differing between poultry farms. Additionally, it was not feasible to merge the passive poultry surveillance data with the active poultry surveillance data due to differences in their respective sampling criteria.

### Potential risk factors

A range of potential spatial-environmental and species-specific AI risk factors were selected for the analyses (Table 1). For the passive wild bird surveillance and the active poultry surveillance, exact coordinates were available for each sample, allowing us to calculate “distance to wetlands” and “distance to coast.” Previous work has shown that the mean T_90_ (time required for a 90% reduction in virus infectivity) of LPAIV (H4N6, H5N1, and H6N8) in lake sediment contaminated by infected wild birds was 2.5 months (21). Additionally, based on laboratory experiments, H5 HPAIV was less persistent than wild-type LPAIV (22), and we, therefore, calculated the distance between sampled poultry farms and the nearest location of a wild bird AIV detection (identified through passive surveillance) within the previous 3 months. Other factors assessed in the analysis of poultry were species, season, flock size, and factors in the analysis of wild birds were order, season, and land cover type.

**Table 1:** Overview of selected risk factors for analysis.

Active poultry surveillance	Passive wild bird surveillance
Species	Order
Flock size	Season
Distance to coast	Land cover type
Distance to wetlands	Distance to coast
Distance to nearest positive wild birds^a^	Distance to wetlands

a Distance to the nearest AIV-positive wild bird collected from the passive wild bird surveillance within the previous 3 months.

We calculated spatial attributes for the sampling locations in R 4.1.2 (23) using Corine Land Cover data and the Danish Map Supply (24, 25). We used a coarse resolution (level 1 classification provided by Corine) to identify the land cover types: artificial surfaces, agricultural areas, forest and semi-natural areas, wetlands, and water bodies. We suspected that temporal patterns of AIV occurrence would reflect the seasonality of wild bird presence, and therefore wanted to include seasonality in our analyses. For the passive wild bird surveillance data, we divided the year into four quarters beginning with January. These four quarters then represented the four seasons over the one-year epidemic period in our study. To reduce the number of classes used when dealing with wild bird species, we aggregated the species according to taxonomic order.

### Statistical analysis

We used descriptive statistics to examine the characteristics of AI occurrence in flocks/events against predictor variables. The statistical model for poultry used surveillance data consisting of test results from all flocks sampled during the study period. However, since different flocks may be situated in the same farm, their test results were likely not independent. In order to address this limitation, we included the CHR number as a random effect in our model. Similarly, because in some cases the same flock was tested multiple times within the same month, we included a random effect of month to account for the temporal correlation. Therefore, for poultry surveillance, we used generalized linear mixed effect models (GLMM) with the crossed random effects CHR number and month. We first performed univariable analyses for each potential risk factor of AI occurrence, and then selected factors with *p*-value < 0.2 to include in a multivariable analysis. This was done using the lme4 package in R 4.1.2 (23, 26). Passive wild bird surveillance consisted of dead birds reported at random unique locations. For the analysis of the wild bird surveillance data, we used simple generalized linear models (GLM) to examine the associations between each potential risk factor and AI occurrence, followed by a multivariable analysis with selected risk factors (*p*-value < 0.2). We also implemented Tukey *post hoc* comparison with Holm adjusted *p*-values to identify significant multi-level risk factors for the GLM (27). The significant threshold for identifying risk factors in the multivariable models of poultry and passive wild bird surveillance was set at *p*-values <0.05.

In the multivariable analyses, we used backward stepwise elimination based on the Akaike Information Criteria (AIC) to obtain the final models with the lowest AIC for the model of each data set. Model estimates and the 95% confidence intervals for significant predictor variables were calculated as the natural logarithm differences of the ORs between each level and the reference level. The log-ORs were back-transformed to ORs using the exponential function.

To explore the overall spatial autocorrelation in residuals of final models for the two types of surveillance data, we first used Moran’s I index in the spdep package in R 4.1.2 (23, 28). This index can evaluate whether spatial locations are significantly clustered, dispersed, or random (29). Notably, wild bird surveillance reported dead birds of different orders at the same coordinates if they were found very close together, but two wild bird events most likely would not be at the exact same location. Thus, we adjusted their positions marginally using the jitter function in R to avoid overlapping points, in order to be able to evaluate any potential spatial autocorrelation. Secondly, we fitted spline (cross)-correlograms of residuals using the R package ncf (30). Correlograms are graphical representations of spatial autocorrelation between locations at a range of lag distances, indicating if spatial correlation is significant at the 95% level.

## Results

### Population and distribution of samples

**Figure 2:**
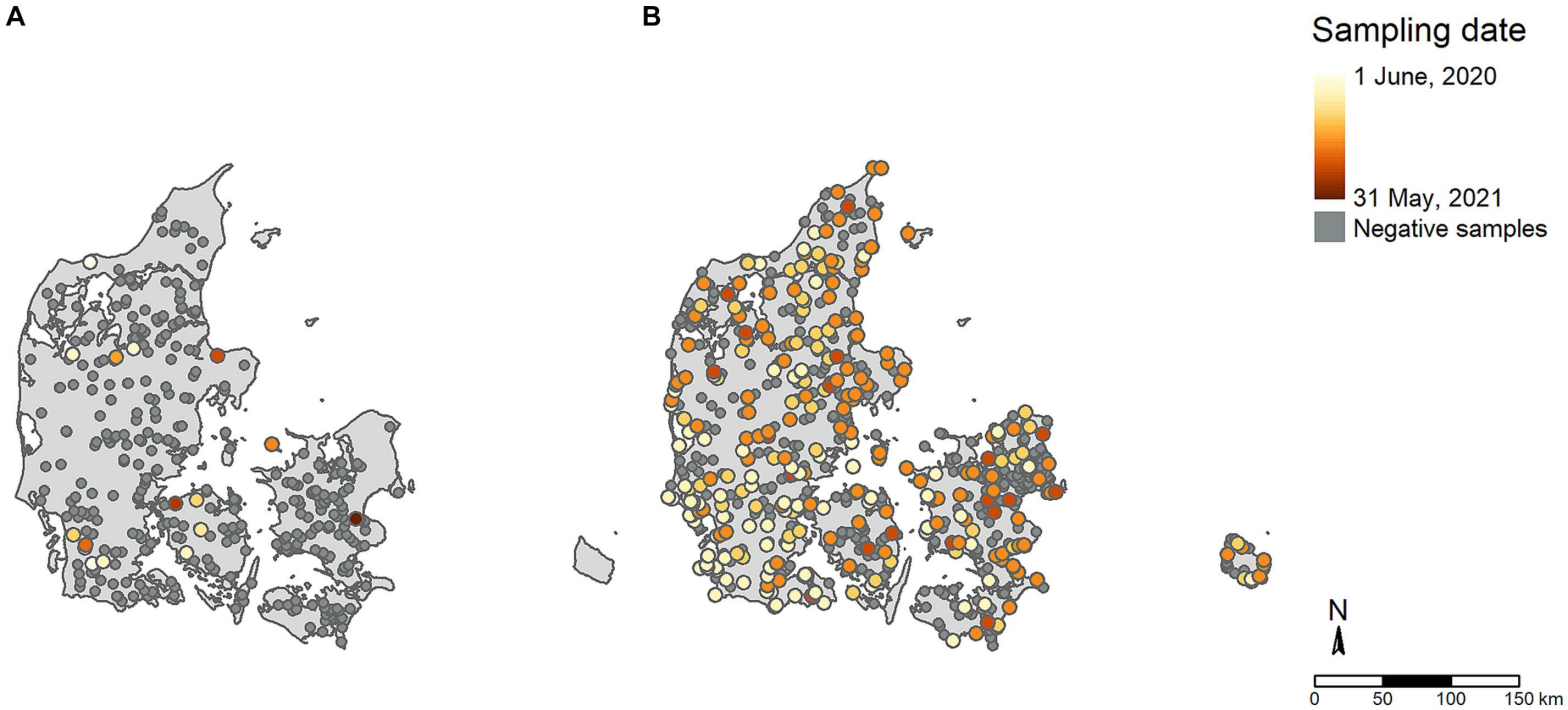
Distribution of samples included in the study. **(A)** Active poultry surveillance; **(B)** Passive wild bird surveillance. Colors denote the chronological order of AI positive cases within the study period, with light colors indicating early periods and dark colors indicating later periods. Samples testing negative for AI are shown in dark grey.

The two original datasets included 11,477 samples from poultry and 852 samples from wild birds tested via passive surveillance. These original samples were further aggregated into 1,062 poultry flock data points and 778 wild bird events from the passive surveillance (Figure 2). Figure 2A shows aggregated AI antibody detections in poultry and virus detections in the offspring of farmed game birds (i.e., farmed mallards, pheasants, and partridges). Figure 2B shows aggregated AIV detections in wild birds.

### Descriptive statistics

**Figure 3:**
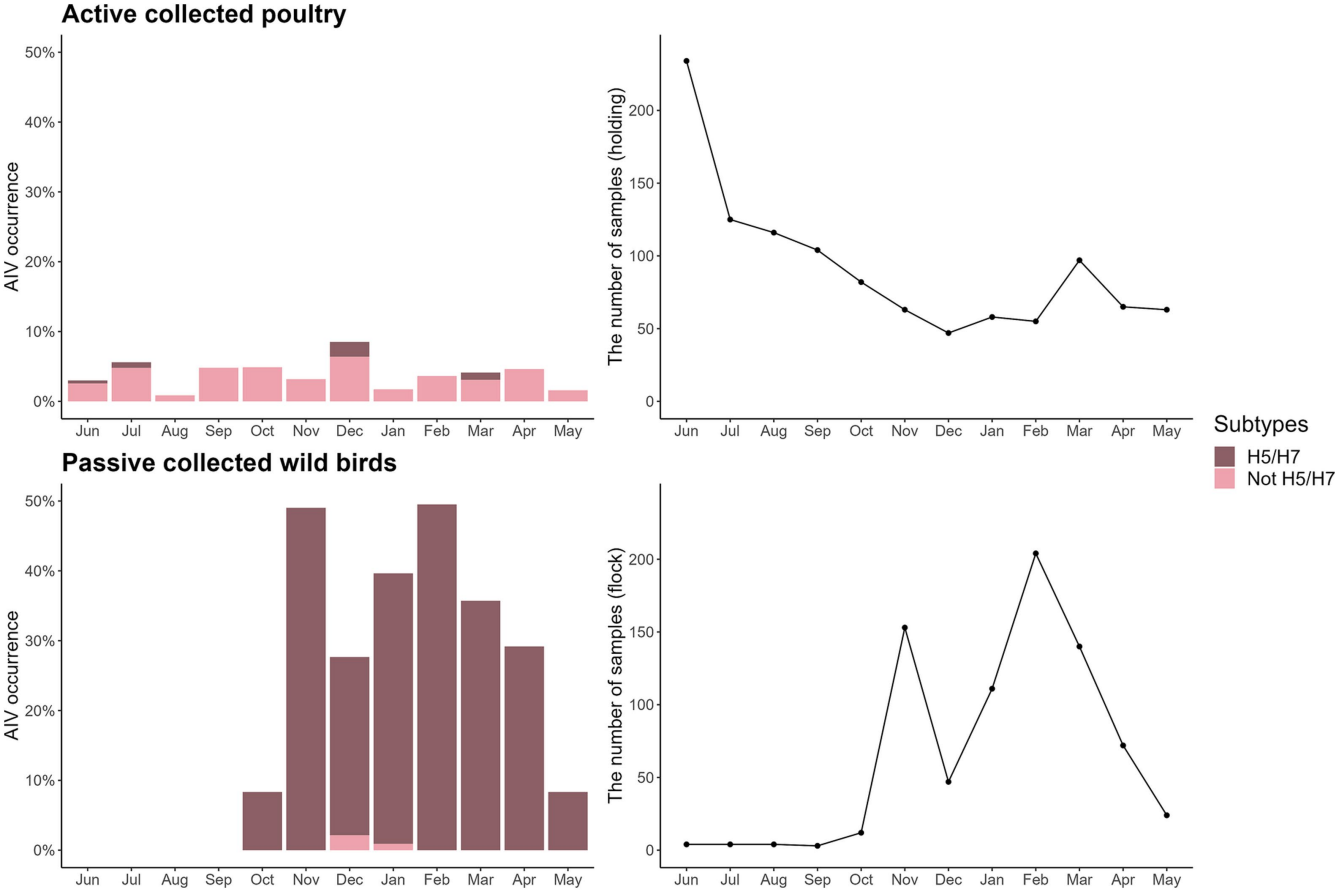
AI occurrence and H5/H7 subtypes identified in the surveillance data (shown as % of all samples within each surveillance type; left column), and the number of samples in actively surveyed poultry and passively surveyed wild birds (right column) during the epidemic wave from June 2020 to May 2021.

For the active poultry surveillance data, 423 Danish poultry farms (unique species and CHR number) were sampled at least once between June 2020 and May 2021, with seven species covered: hens/chickens, domestic ducks, turkeys, geese, farmed mallards, farmed pheasants, and farmed partridges. The distance from the sampled farms to the closest wetland ranged from 0.07 to 21 km (median: 3.9 km), and the distance to the closest coast ranged from 0.27 to 50 km (median: 14 km). Furthermore, the distance to the nearest AIV-positive wild bird collected from the passive wild bird surveillance within the previous 3 months ranged from 0.19 to 302 km (median: 21 km). A total of 187 out of 1,062 poultry flock samples were excluded when analysing the effect of distance to the nearest AIV-positive wild bird, because no dead AIV-positive wild birds were found within 3 months of the farm sampling date (mainly between June and September) (Figure 3). For poultry, 39 of the 1,062 (4%) samples were AI positive, 4 (10%) of which were H5/H7 subtypes (Supplementary Table 1). The peak in poultry submission observed in June and July (Figure 3), was caused by testing of game bird offspring. Game bird offspring were tested for AIV before being released at 3–6 weeks of age, the number of which was most frequent in June and July.

Samples from 60 bird species were collected in the passive wild bird surveillance, covering 13 bird orders (Anseriformes, Galliformes, Accipitriformes, Charadriiformes, Podicipediformes, Suliformes, Gruiformes, Columbiformes, Passeriformes, Piciformes, Pelecaniformes, Strigiformes, and Falconiformes). These birds were found in five land cover types. Distance to the closest wetland ranged from 0.0019 to 23 km (median = 3.8 km), and distance to the closest coast ranged from 0.043 to 50 km (median = 8.8 km). The passive wild bird surveillance data consisted of 471 AIV-negative and 307 (39%) AIV-positive detections, with the latter consisting of 305 (99%) records of H5/H7 subtypes (Figure 3).

The most often identified subtype in dead wild birds (passive surveillance) was H5N8. Results of subtypes in active poultry surveillance were dominated by antibodies not belonging to H5/H7. AIV-positive game bird offspring were not infected with H5 or H7 subtypes.

The proportion of virus subtypes detected in the data varied across surveillance approaches (i.e., active or passive; Table 2). Furthermore, monthly variation in the number of samples was evident in wild birds, with sparse sampling and no AIV-positive birds detected from June to September.

**Table 2:** Subtyping results for AIV-positive detected samples during June 2020 and May 2021^a^.

	Active poultry surveillance	Passive wild bird surveillance
H5N1 HPAI virus		3
H5N3 HPAI virus		2
H5N5 HPAI virus		6
H5N8 HPAI virus		291
H5Nx^b^ HPAI virus		5
Not H5 or H7 virus	3	2
H5 antibody	4	
H7 antibody	0	
Not H5 or H7 antibody	32	

a In some aggregated samples from the same poultry flock or location of wild bird collections, several subtypes were detected in the same aggregation.

b NA-subtype was not determined.

### Univariable analyses

The univariable GLMM models did not identify significant (*p*-value < 0.05) risk factors in the active poultry surveillance data (Supplementary Table 2). In the wild bird surveillance data, the GLM identified the bird order Anseriformes (e.g., geese and ducks) to have a higher risk of AI detection than Accipitriformes (e.g., Eurasian sparrowhawk) and Charadriiformes (e.g., gulls).

**Figure 4:**
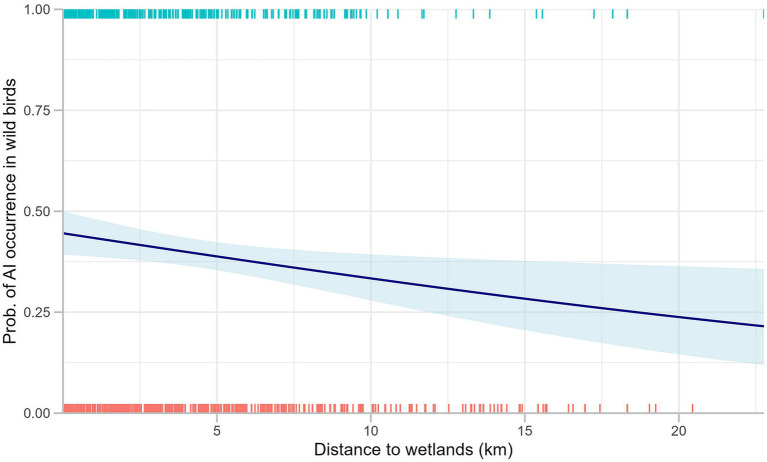
Distribution of AI occurrence in passively surveyed wild birds against distance to wetlands. Marginal rugs represent the binary surveillance results, and the blue line displays a fitted curve for binary logistic regression between two variables.

In addition, we found a significantly higher risk of AI detection during the spring compared to summer for passively surveyed wild birds. The risk of AI occurrence decreased significantly with an increasing distance to wetlands for passively surveyed wild birds. A rug plot shows the distribution of AI occurrence found in passive wild bird surveillance, and the decrease in the probability of AI occurrence with the increased distance to wetlands (Figure 4). As none of the variables in the models for active poultry surveillance exhibited *p*-values below 0.2, no variables were eligible for inclusion in a multivariable analysis. In the passive wild bird surveillance, we selected distance to wetlands, bird order, season, and land cover type for the full multivariable model.

After failing to include any potential risk factors into the poultry multivariable model, we examined the spatial autocorrelation of residuals from the univariable models for the poultry surveillance data. Moran’s I statistics indicated that the spatial autocorrelation of residuals of univariable models for active poultry surveillance was not significant (distance to coast: I = −0.61, *p* = 1; distance to wetlands: I = −0.6, *p* = 1; flock size: I = −0.6, *p* = 1; species: I = 0.59, *p* = 1; Distance to nearest positive wild birds: −0.67, *p* = 1).

### Multivariable analysis

**Figure 5:**
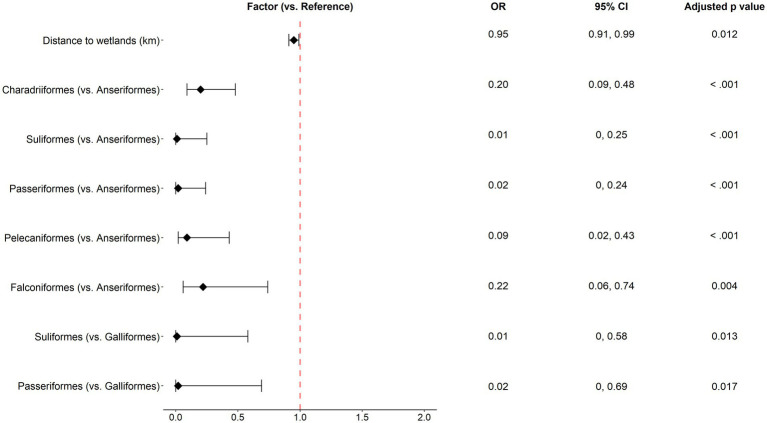
Odds ratios with 95% confidence intervals for the significant factors identified in the multivariable analysis of wild bird passive surveillance data. Statistical significance was denoted by Tukey post-hoc comparisons (Holm-adjusted *p* values <0.05).

For passively surveyed wild birds, for every 1 km increase in distance to wetlands, the risk of AIV occurrence decreased by 5.18% (OR 0.95), when other variables remained the same. The order Anseriformes had a high risk of AIV occurrence compared to Pelecaniformes, Charadriiformes, Falconiformes, Suliformes, and Passeriformes. The order Galliformes was associated with a higher risk than Suliformes and Passeriformes (Figure 5).

The goodness of fit (*R*^2^) for the multivariable model was 0.21 (Table 3), showing relatively poor predictive power, similar to previous studies (16). For the final wild bird surveillance model, Moran’s I indicated significant spatial dependence among residuals, suggesting surveillance locations deviated from a random distribution of sampling locations (I = 0.19, *p* < 0.001). However, upon closer examination of spline (cross)-correlograms of the final model residuals, the spatial dependence envelope overlapped with zero across the whole scale (Supplementary Figure 1). This observation indicates a weak autocorrelation between neighboring locations over distance.

**Table 3:** The final multivariable model for AI detection using the passive wild bird surveillance data.

Factor	*p*-value
Distance to wetlands	0.012*
Land cover type	0.072
Order	<0.001*

* Significance level, *p* < 0.05 by Chi-square analysis.

## Discussion

The objective of the study was to identify risk factors associated with AI in wild and domestic birds by use of data from active surveillance in poultry and passive surveillance in wild birds. Our results revealed that when accounting for spatial and temporal components in the active poultry surveillance, we were not able to identify any significant risk factors, whereas for passive surveillance of wild birds there was a higher risk of the bird order Anseriformes being AIV positive, and a decreasing risk with increasing distance to wetlands. Although domestic ducks were not found to have higher risks than the other domestic species in our study using active surveillance data, other studies have shown that ducks are very susceptible to AIV and resistant to virus-induced mortality (specifically 2016 H5N8 Clade 2.3.4.4b) but can efficiently transmit viruses to other birds such as turkeys (31). In 2020, EU member states reported that the proportion of H5/H7 seropositive poultry establishments was 1.8% for breeding ducks, whereas it was below 1% in all other poultry categories (4). For wild birds, an experimental study found that H5N1 mortality in mallards ranged from 0 to 100% depending on the genotype, underlining the risk of mallards acting as a silent reservoir for H5N1 viruses (32). Furthermore, in Europe, mute and whooper swans can act as potential sentinels due to their high susceptibility to HPAI and the swans being conspicuous when they die (33, 34).

In general, land cover type was not significantly associated with AIV detection in our analyses, but we did find significant associations with distance to wetlands from passively collected birds. This may indicate that the passive collection of dead birds by authorities would benefit from additional focus on water body areas. This finding was in line with what has previously been found in Denmark, where analyses of 15 years of wild bird surveillance showed that decreasing distance to wetlands was positively associated with AIV presence (16).

In the Netherlands, the risk of LPAIV introduction in poultry farms decreased significantly with increasing distance to medium-sized waterways, and the median distance for all investigated farms was often less than 1 km (35). For the actively sampled poultry farms in this study, the median distance to wetlands was 3.9 km and the median distance to coastline was 14.3 km, which was much further than the Dutch cases. This difference might lead to less significant effects of distance to waterways in our analysis. The difference in farm selection was also evident, when we compared their significant effect of distance to wild bird areas (with a median distance of less than 5 km) to our analysis, where we did not find a statistically significant effect of distance between poultry farms and the nearest location of a wild bird AIV case (with a median distance of 21 km).

A clear effect of seasonality has been observed in HPAI detections recorded by the World Organisation for Animal Health (WOAH) for 199 countries, with the lowest number of detections in September, a rise in October, and a peak in February (36). In our multivariable models for wild birds, season also showed a significant association with AIV occurrence. However, a bias in the data for passive surveillance is that the Danish authorities stop collecting dead birds at a distance of 20 km from a known positive case for 4 weeks, which might have lowered the detection rate in AI intensive period compared to the less intensive period.

Flock size is often found to be a risk factor for infectious disease and outbreak occurrence in farmed animals (37, 38). However, we did not find a significant association between poultry flock size and AIV occurrence in active surveillance data. In the poultry system, contact with wild birds or contaminated fomites, and biosecurity have previously been identified as risk factors by summarising the analysis of 54 AI introductions in European poultry farms (39). Furthermore, the distance from a poultry farm to the closest AIV-positive wild bird within the previous 3 months was not a significant risk factor for the occurrence of AI in poultry flocks in our study. This was surprising, as we expected proximity to AIV-positive wild birds to be an indicator of AIV presence in the area. Belkhiria et al. (40) observed the association between AI incidence in poultry and wild bird abundance during the migratory season in the USA, indicating that wild birds migrating from other countries or regions introduced disease to farms. Li et al. (41) also found a high degree of spatiotemporal overlap between the core pathway area of migratory whooper swans and historical HPAIV H5N1 wild bird events in China and Mongolia between 2005 and 2015, indicating that swans could carry HPAI H5N1 virus during migration, resulting in long-distance transmission. However, the exclusion of passive poultry surveillance data (i.e., outbreak detections based on clinical signs) may influence the identification of significant risk factors, especially regarding distance from wild birds. In addition, active poultry surveillance was risk-based, focussing on herd types either considered with a higher risk (outdoor farming) or potentially constituting a higher risk by selling poultry. It is generally difficult to detect outbreaks using active surveillance, especially for HPAI which spread rapidly once introduced to flocks (42). The non-significant effect of distance to detection in wild birds may also be explained by the lack of information for the distance variable in 187 records from our poultry data (i.e., not knowing the presence/absence of diseased wild birds around the actively surveyed farms), wherein ten of them were tested AI positive. Also, the contrast between high (99%) H5/H7 occurrence in positive, passive surveyed wild birds and relatively low (10%) H5/H7 occurrence in active surveyed poultry suggested poultry farms in Denmark might explained by the housing rules, which is a preventive biosecurity measure against HPAI introduction from positive wild birds. In addition to a suspected overlap in outbreaks in wild and poultry birds, it would also be worth investigating dominating AIV subtypes and thus obtain further knowledge about disease transmission by investigating the evolutionary relationships of viral strains.

Other studies have found high bird activity and AIV survival rate to be associated with low temperatures and high humidity. For example, viral persistence was significantly higher on duck feather samples at 10°C compared to 25°C and 37°C (43), which could further explain the low occurrence of AIV detection in wild birds during the warmer summer months in Denmark.

Our model could be improved by including other potential risk factors, using more information and updated surveillance data. Anthropogenic variables may have been responsible for the initial introduction to poultry farms, which may mask the association between some of our selected risk factors and AIV detection. Certain management factors, such as having multiple egg production sites, were found to be risk factors for the introduction of AIV to poultry farms in France (44). For wild birds, it has been suggested that urbanization and deforestation transforming wild bird habitats could contribute to altered flyways and increased contact between different wild bird populations (45). In Denmark, the populations of different water bird species vary. For example, the population size of barnacle geese in Denmark has increased five-fold in recent decades, while the size of the Danish mallard population is relatively stable (46, 47). Bird migration flyways, wild bird densities, and bird movement are all suspected to play a role in AI transmission and should be considered when investigating the seasonality of AI detection. In addition, there are suspected relationships between eco-climatic variables and the risk of AI occurrence. However, these variables include many elements (e.g., temperature, precipitation, and vegetation coverage), and the aggregation level (e.g., monthly or daily) makes it difficult to use them for prediction purposes (48).

The data sets used in this study were not designed for analyses representing overall bird populations in Denmark. There may, therefore, be some sampling bias when reporting AIV cases in wild birds, which may have led to geographical and taxonomic bias. Kjær et al. (16) investigated accessibility bias in the Danish passive surveillance data. They found that a geographical bias existed, as most of the recorded locations in the passive surveillance data were within 35 km of larger cities and within 500 m of roads, and that numerous records were close to the coast potentially due to public access to beaches in Denmark (16). Another aspect linked to the original data sets is the differences between the aims of the two surveillance programs. Active surveillance for poultry targeted virus circulation, implying detection of mostly LPAIV (or non-H5/H7) strains on farms due to the high mortality of HPAIV strains; in contrast, passive surveillance of wild birds tended to detect the introduction/circulation of HPAIV before farm outbreaks (Table 2). This also explained why we could not find a closer detection of a wild bird event being related to AI occurrence in actively surveyed poultry farms. For passive wild bird surveillance, Moran’s I indicated significant spatial dependence among locations, while the spline correlograms indicated that the autocorrelation was weak. These results suggest that inclusion of other spatial factors may improve the model.

Many modeling approaches have been implemented in spatiotemporal analyses of AIV. For example, boosted regression trees (BRT) allow for a gradual fitting process. A previous study used BRT models and logistic regressions to test for associations between poultry production structure and H5N1 risk in Thailand (49). The authors found that the BRT had a goodness of fit that was better than or comparable to that of logistic regression when predictions were evaluated by different data sets. However, evidence of over-fitting for BRT was noted in other studies (50). Another modeling approach focused on the animal/case level and used spatial regression models with Bayesian inference under zero-inflated Poisson regression (51). The authors found a negative association between the density of chickens and outbreak risk due to high vaccine coverage. However, our study is at the population level and disregards information about the number of birds in one flock or event. Another aspect of AI spread is human-mediated transmission between holdings. This can be analyzed using network analysis to quantify human-mediated risks such as the movement of live poultry (52). However, it would require more farm-level data, including human behavior data, which are not currently available in the Danish surveillance data.

For the last 3 years, HPAI H5 viruses have been responsible for AI outbreaks in the Danish poultry sector. However, in this study, we included all AI detections regardless of subtype (thus including both LPAI and HPAI) from the assumption that the risk of AI infection in poultry is the same for all subtypes (53, 54). While some virus strains have higher case fatality rates, others with lower case fatality rates might spread more efficiently among wild birds, leading to constant infection pressure from wild birds to poultry. Therefore, we found it reasonable to investigate all types of AIV as a proxy for transmission between wild birds and poultry.

To conclude, we evaluated spatiotemporal risk factors for AI detection in Danish poultry and wild bird populations using Danish national surveillance data and found associations between potential risk factors and AI occurrence during an epidemic wave. Overall, bird orders and distance to wetlands were associated with the occurrence of AI. The findings present potential targets for surveillance strategies using limited resources and aid risk-based surveillance during epidemics.

## Data availability statement

The original contributions presented in the study are included in the article/Supplementary material, further inquiries can be directed to the corresponding author.

## Author contributions

YL: Conceptualization, Data curation, Formal analysis, Investigation, Methodology, Software, Validation, Visualization, Writing – original draft, Writing – review & editing. LK: Formal analysis, Investigation, Methodology, Supervision, Validation, Writing – review & editing. AB: Formal analysis, Investigation, Methodology, Supervision, Validation, Writing – review & editing. CH: Methodology, Validation, Writing – review & editing. LL: Methodology, Validation, Writing – review & editing. CK: Formal analysis, Funding acquisition, Investigation, Methodology, Project administration, Supervision, Validation, Writing – review & editing.

## Footnotes

^1^ https://www.retsinformation.dk/eli/lta/2019/1456
