# Supplementary material for: Risk factors for avian influenza in Danish poultry and wild birds during the epidemic from June 2020 to May 2021
Source: Front Vet Sci. 2024 Feb 21;11:1358995. doi: 10.3389/fvets.2024.1358995 (PMC10914952; doi:10.3389/fvets.2024.1358995)
Supplement: Supplementary file 1 [file Data_Sheet_1.docx]

**Supplementary material:** AI surveillance in Denmark (with more laboratory details)

Active poultry serological surveillance was performed in 2020 and 2021. Blood samples from apparently healthy birds in poultry farms with more than 100 animals were tested for influenza A virus antibodies using an enzyme-linked immunosorbent assay (ELISA). ELISA-positive samples were tested for H5/H7 using haemagglutination inhibition (HI) test against H5 and H7 reference antigens provided by the European Reference Laboratory (EURL) for AI. Furthermore, ELISA-positive samples from poultry belonging to the Anatidae family (mostly domestic ducks and geese) were also tested against clade 2.3.4.4 H5 antigens from the EURL for AI. When H5/H7 antigens were detected, tests for circulating H5/H7 viruses were conducted using reverse transcriptase polymerase chain reaction (RT-PCR) tests, typically within 5–10 days after blood sample collection. Moreover, a national surveillance program for the CHR registered offspring of game birds raised for restocking (i.e., farmed mallards, farmed pheasants, and farmed partridges) was performed using RT-PCR testing for the virus.

Wild birds found dead in the environment were tested as part of the EU mandatory passive surveillance for AI. Each bird was tested by RT-PCR for the presence of AIV in a pool of a cloacal and a tracheal swabs). In addition, brain tissue swabs from each bird were also routinely tested from the end of January 2021.

RT-PCR detection, H5/H7 subtyping, and pathotyping by sequencing were performed as previously described (1).

The tested wild birds included mainly species considered to be AI high-risk target species (2).

References

1. Liang Y, Krog JS, Ryt-Hansen P, Pedersen AG, Kvisgaard LK, Holm E, et al. Molecular Characterization of Highly Pathogenic Avian Influenza Viruses H5N6 Detected in Denmark in 2018–2019. Viruses. 2021;13(6).

2. European Food Safety Authority, European Centre for Disease Prevention and Control, European Union Reference Laboratory for Avian influenza, Brown I, Kuiken T, Mulatti P, et al. Avian influenza overview September – November 2017. EFSA Journal. 2017 Dec 1;15(12):e05141.
