# Supplementary material for: Risk factors for avian influenza in Danish poultry and wild birds during the epidemic from June 2020 to May 2021
Source: Front Vet Sci. 2024 Feb 21;11:1358995. doi: 10.3389/fvets.2024.1358995 (PMC10914952; doi:10.3389/fvets.2024.1358995)
Supplement: Supplementary file 2 [file Image_1.pdf]

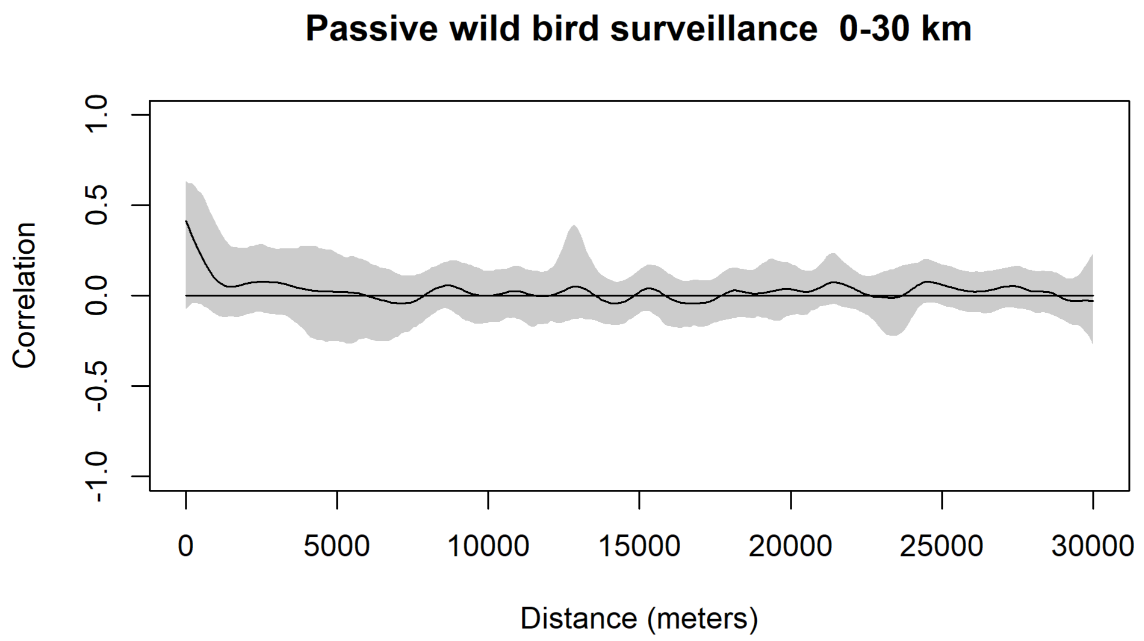

Supplementary Figure 1. Spline (cross)-correlograms of residuals of the final wild bird model, presenting spatial autocorrelation at distances up to 30 kilometers with 95% confidence intervals.
