# Supplementary material for: Risk factors for avian influenza in Danish poultry and wild birds during the epidemic from June 2020 to May 2021
Source: Front Vet Sci. 2024 Feb 21;11:1358995. doi: 10.3389/fvets.2024.1358995 (PMC10914952; doi:10.3389/fvets.2024.1358995)
Supplement: Supplementary file 3 [file Table_1.docx]

**Supplementary Table 1.** Descriptive analysis for actively surveyed poultry and passively surveyed wild birds from 1 June 2020 to 31 May 2021.

| Active surveillance in poultry | | | | |
| --- | --- | --- | --- | --- |
| **Variables** | **Level** | **n (%)** | **AI- (%)** | **AI+ (%)** |
| **Overall** |  | 1,062 | 1023 (96%) | 39 (4%) |
| **Species** | Domestic ducks | 41 (4%) | 32 (78%) | 9 (22%) |
|  | Geese | 9 (1%) | 8 (89%) | 1 (11%) |
|  | Farmed mallard | 43 (4%) | 37 (86%) | 6 (14%) |
|  | Hens/chickens | 684 (64%) | 664 (97%) | 20 (3%) |
|  | Farmed partridges | 14 (1%) | 14 (100%) | 0 (0%) |
|  | Farmed pheasants | 238 (22%) | 236 (99%) | 2 (1%) |
|  | Turkeys | 33 (3%) | 32 (97%) | 1 (3%) |
| **Season** | Spring | 209 (20%) | 202 (97%) | 7 (3%) |
|  | Summer | 334 (31%) | 325 (97%) | 9 (3%) |
|  | Autumn | 334 (31%) | 319 (96%) | 15 (4%) |
|  | Winter | 190 (18%) | 177 (93%) | 13 (7%) |
| **Flock size** | Small (0, 100] | 83 (8%) | 78 (94%) | 5 (6%) |
|  | Medium–small (100, 1,000] | 296 (28%) | 285 (96%) | 11 (4%) |
|  | Medium (1,000, 10,000] | 230 (22%) | 225 (98%) | 5 (2%) |
|  | Median–large (10,000, 100,000] | 434 (41%) | 416 (96%) | 18 (4%) |
|  | Large (100,000, 300,000] | 19 (2%) | 19 (100%) | 0 (0%) |
| **Dcoast (km)** | Very short (0, 6.92] | 266 (25%) | 253 (95%) | 13 (5%) |
|  | Short (6.92, 14.32] | 263 (25%) | 252 (96%) | 11 (4%) |
|  | Far (14.32, 21.4] | 272 (26%) | 268 (99%) | 4 (1%) |
|  | Very far (21.4, 50.41] | 261 (25%) | 250 (96%) | 11 (4%) |
| **Dwetland (km)** | Very short (0, 2.16] | 269 (25%) | 258 (96%) | 11 (4%) |
|  | Short (2.16, 3.94] | 264 (25%) | 257 (97%) | 7 (3%) |
|  | Far (3.94, 5.66] | 265 (25%) | 259 (98%) | 7 (3%) |
|  | Very far (5.66, 21.15] | 264 (25%) | 249 (94%) | 15 (6%) |
| **Dwild (km)** | Very short (0, 9.77] | 219 (25%) | 214 (98%) | 5 (2%) |
|  | Short (9.77, 20.77] | 219 (25%) | 208 (95%) | 11 (5%) |
|  | Far (20.77, 46.01] | 218 (25%) | 209 (96%) | 9 (4%) |
|  | Very far (46.01, 301.65] | 219 (25%) | 214 (98%) | 5 (2%) |
|  |  |  |  |  |
| Passive surveillance in wild birds | | | | |
| **Variables** | **Level** | **n (%)** | **AI- (%)** | **AI+ (%)** |
| **Overall** |  | 778 | 471 (60%) | 307 (40%) |
| **Order** | Anseriformes | 256 (33%) | 84 (33%) | 172 (67%) |
|  | Galliformes | 8 (1%) | 4 (50%) | 4 (50%) |
|  | Accipitriformes | 231 (30%) | 151 (65%) | 80 (35%) |
|  | Charadriiformes | 95 (12%) | 68 (72%) | 27 (28%) |
|  | Podicipediformes | 1 (0%) | 1 (100%) | 0 (0%) |
|  | Suliformes | 47 (6%) | 46 (98%) | 1 (2%) |
|  | Gruiformes | 13 (2%) | 10 (77%) | 3 (23%) |
|  | Columbiformes | 2 (0%) | 2 (100%) | 0 (0%) |
|  | Passeriformes | 48 (6%) | 46 (96%) | 2 (4%) |
|  | Piciformes | 1 (0%) | 1 (100%) | 0 (0%) |
|  | Pelecaniformes | 30 (4%) | 24 (80%) | 6 (20%) |
|  | Strigiformes | 11 (1%) | 11 (100%) | 0 (0%) |
|  | Falconiformes | 35 (4%) | 23 (66%) | 12 (34%) |
| **Season** | Spring | 455 (58%) | 260 (57%) | 195 (43%) |
|  | Summer | 100 (13%) | 77 (77%) | 23 (23%) |
|  | Autumn | 11 (1%) | 11 (100%) | 0 (0%) |
|  | Winter | 212 (27%) | 123 (58%) | 89 (42%) |
| **Land cover type** | Artificial surfaces | 316 (41%) | 208 (66%) | 108 (34%) |
|  | Agricultural areas | 300 (39%) | 176 (59%) | 124 (41%) |
|  | Forest and semi-natural areas | 81 (10%) | 41 (51%) | 40 (49%) |
|  | Wetlands | 32 (4%) | 19 (59%) | 13 (41%) |
|  | Water bodies | 49 (6%) | 27 (55%) | 22 (45%) |
| **Dcoast (km)** | Very short (0, 4.36] | 195 (25%) | 114 (58%) | 81 (42%) |
|  | Short (4.36, 8.83] | 195 (25%) | 123 (63%) | 72 (37%) |
|  | Far (8.83, 16.5] | 195 (25%) | 117 (60%) | 78 (40%) |
|  | Very far (16.5, 49.6] | 193 (25%) | 117 (61%) | 76 (39%) |
| **Dwetland (km)** | Very short (0, 1.55] | 196 (25%) | 95 (48%) | 101 (52%) |
|  | Short (1.55, 3.5] | 194 (25%) | 127 (65%) | 67 (35%) |
|  | Far (3.5, 6.5] | 196 (25%) | 128 (65%) | 68 (35%) |
|  | Very far (6.5, 22.8] | 192 (25%) | 121 (63%) | 71 (37%) |
