# Supplementary material for: Risk factors for avian influenza in Danish poultry and wild birds during the epidemic from June 2020 to May 2021
Source: Front Vet Sci. 2024 Feb 21;11:1358995. doi: 10.3389/fvets.2024.1358995 (PMC10914952; doi:10.3389/fvets.2024.1358995)
Supplement: Supplementary file 4 [file Table_2.docx]

**Supplementary Table 2.** Univariable binomial regression (odds ratios) models of AIV occurrence for the active poultry surveillance data and the passive wild bird surveillance data.

| **Surveillance data** | **Levels** | **OR** | **95% CI** | **p-value** |
| --- | --- | --- | --- | --- |
| **Active poultry surveillance:** |  |  |  |  |
| Distance to coast (km) | (cont.) | 0.97 | 0.67–1.41 | 0.88 |
| Distance to wetlands (km) | (cont.) | 1.09 | 0.78–1.53 | 0.60 |
| Distance to positive wild birds (km) | (cont.) | 0 | 0–0 | 0.29 |
| Flock size | (cont.) | 1 | 1–1 | 1 |
| Species |  |  |  | 0.44 |
|  | Domestic ducks | Ref. |  |  |
|  | Geese | 0 | 0–0.39 | 0.04 |
|  | Farmed mallards | 0.38 | 0–3651.48 | 0.84 |
|  | Hens/chickens | 0.03 | 0–68.58 | 0.37 |
|  | Farmed partridges | 0.01 | 0–1.07E+49 | 0.94 |
|  | Farmed Pheasants | 0 | 0–204.15 | 0.32 |
|  | Turkeys | 0.17 | 0–2766.61 | 0.72 |
| **Passive wild bird surveillance** |  |  |  |  |
| Distance to coast (km) | (cont.) | 1.0061 | 0.9913–1.021 | 0.419 |
| Distance to wetlands (km) | (cont.) | 0.9539 | 0.9181–0.9897 | 0.012 |
| Season |  |  |  | <0.001 |
|  | Spring | Ref. |  |  |
|  | Summer | 0.3983 | 0.2367–0.6479 | <0.001 |
|  | Autumn |  |  | 0.972 |
|  | Winter | 0.9648 | 0.6927–1.3408 | 0.831 |
| Order |  |  |  | <0.001 |
|  | Anseriformes | Ref. |  |  |
|  | Galliformes | 0.4884 | 0.113–2.1107 | 0.319 |
|  | Accipitriformes | 0.2587 | 0.1769–0.3756 | <0.001 |
|  | Charadriiformes | 0.1939 | 0.1142–0.3217 | <0.001 |
|  | Podicipediformes |  |  | 0.994 |
|  | Suliformes | 0.0106 | 0.0006–0.0499 | <0.001 |
|  | Gruiformes | 0.1465 | 0.0322–0.4934 | 0.004 |
|  | Columbiformes |  |  | 0.992 |
|  | Passeriformes | 0.0212 | 0.0034–0.0709 | <0.001 |
|  | Piciformes |  |  | 0.994 |
|  | Pelecaniformes | 0.1221 | 0.0439–0.292 | <0.001 |
|  | Strigiformes |  |  | 0.981 |
|  | Falconiformes | 0.2548 | 0.1174–0.5277 | <0.001 |
| Land cover type |  |  |  | 0.089 |
|  | Artificial surfaces | Ref. |  |  |
|  | Agricultural areas | 1.3569 | 0.979–1.8832 | 0.067 |
|  | Forest and semi-natural areas | 1.8790 | 1.1458–3.0833 | 0.012 |
|  | Wetlands | 1.3177 | 0.6142–2.7488 | 0.467 |
|  | Water bodies | 1.5693 | 0.8473–2.8818 | 0.147 |

Ref.: reference categories; Cont.: continuous variables.
